# The Growth Hormone Secretagogue Receptor: Its Intracellular Signaling and Regulation

**DOI:** 10.3390/ijms15034837

**Published:** 2014-03-19

**Authors:** Yue Yin, Yin Li, Weizhen Zhang

**Affiliations:** 1 Department of Physiology and Pathophysiology, Peking University Health Science Center, Beijing 100191, China; E-Mails: bingningxingwan@sina.com (Y.Y.); yinli@bjmu.edu.cn (Y.L.); 2 Department of Surgery, University of Michigan Medical Center, Ann Arbor, MI 48109-0346, USA

**Keywords:** ghrelin receptor, constitutive activity, intracellular signaling, [Ca^2+^]*_i_*, AMPK, mTOR, MAPK, PI3K

## Abstract

The growth hormone secretagogue receptor (GHSR), also known as the ghrelin receptor, is involved in mediating a wide variety of biological effects of ghrelin, including: stimulation of growth hormone release, increase of food intake and body weight, modulation of glucose and lipid metabolism, regulation of gastrointestinal motility and secretion, protection of neuronal and cardiovascular cells, and regulation of immune function. Dependent on the tissues and cells, activation of GHSR may trigger a diversity of signaling mechanisms and subsequent distinct physiological responses. Distinct regulation of GHSR occurs at levels of transcription, receptor interaction and internalization. Here we review the current understanding on the intracellular signaling pathways of GHSR and its modulation. An overview of the molecular structure of GHSR is presented first, followed by the discussion on its signaling mechanisms. Finally, potential mechanisms regulating GHSR are reviewed.

## 1. Introduction

Growth hormone secretagogue receptor (GHSR), also known as ghrelin receptor, was first identified as the target of the growth hormone secretagogue (GHS) and subsequently cloned in human pituitary and hypothalamus [1]. It is a heterotrimeric G protein-coupled receptor (GPCR) containing 366 amino acids with the typical seven transmembrane domains (TMI-VII). Both the peptidyl (GHRP-6) and nonpeptide (MK-0677) growth hormone secretagogues stimulate growth hormone release through activation of this specific GPCR expressed on the surface of somatotroph in the anterior pituitary gland [2–4]. Its endogenous ligand was identified a few years later from stomach extracts and named ghrelin when Kojima *et al.* used the Chinese hamster ovary cell line expressing the rat GHSR to screen various tissue preparations for the characteristic increase in intracellular calcium concentrations ([Ca^2+^]*_i_*) induced by the GHSs [5].

To date, the physiological functions of GHSR have been extended to include: (1) the release of various hormones such as growth hormone, adrenocorticotropic hormone, cortisol, and prolactin [6]; (2) modulation of food intake and energy metabolism [7]; (3) influences on glucose and lipid metabolism [6]; (4) regulation of gastrointestinal motility and secretion [8], and pancreatic function [9]; (5) regulation of cell proliferation and survival [10,11]; (6) attenuation of proinflammatory cascades and regulation of immune function that play important roles in aging and gastrointestinal homeostasis [4]; and (7) cell protection in the nervous and the cardiovascular systems [12–14]. Such diversified functions of GHSR suggest the complexity of GHSR-mediated intracellular signaling. Numerous intracellular signaling pathways have been proposed upon activation of GHSR. This review summarizes recent advances concerning the intracellular signaling mechanisms of GHSR with a focus on its functional relevance. We will first introduce the molecular structure of GHSR, then discuss in detail its key intracellular signaling mechanisms, and finish with the current understanding on the modulation of GHSR. Although two isoforms of GHSR: 1a and 1b, have been identified, GHSR1a, which is traditionally considered as the active form of GHSR, is the focus of much investigation; we therefore focus our discussion on the GHSR1a.

## 2. Molecular Structure of GHSR

Located on chromosome 3q26.2, the *GHSR* gene encodes two transcripts: 1a and 1b. The *GHSR1a* is encoded by a 1.1 kb noncontiguous open reading frame, which is divided into exon 1 and exon 2 encoding an amino-terminal TM I–V segment and a carboxyl-terminal TM VI/VII segment respectively by an approximate 2 kb of noncoding intron [2,15]. The intron contains a stop codon that may lead to the production of *GHSR1b* mRNA by alternative splicing. Both sequences are identical from the Met translation site to Leu^265^. Over 90% of sequence homology has been found between the predicted human, rat, pig, and sheep GHSR1a amino acid sequences [16]. Human GHSR1a consists of 366 amino acids with a molecular mass of approximate 41 kDa [1]. As a member of GPCRs, GHSR1a contains seven transmembrane α-helix hydrophobic domains connected by three intra- and extracellular domains, beginning with an extracellular *N*-terminal domain and ending with an intracellular *C*-terminal domain [17]. The *N*-terminal domain forms a β-hairpin structure, while the TM domains form a round calyx-like structure with the Pro residues in the center of the TM helices. Among seven TM domains, TM III occupies the central position, while TM V is the most peripheral [18]. TM II and TM III are considered the ligand activation domains. Three conserved residues, Glu^140^-Arg^141^-Tyr^142^, located at the intracellular end of TM III are critical for the isomerization between the active and inactive conformation. Two conserved cysteine residues (Cys^116^ and Cys^198^) on extracellular loops 1 and 2 form a disulfide bond [19,20]. These key amino acid residues, which have been evolutionarily conserved for 400 million years, are essential for binding and activation of GHSR1a by different ligands, highlighting their importance in the physiological processes [16]. GHSR1b contains 298 amino acids corresponding to the first five TM domains encoded by exon 1, plus a unique 24 amino acid tail encoded by an alternatively spliced intronic sequence [2]. GHSR1b neither binds nor responds to ghrelin or GHSs [21]. However, *GHSR1b* gene is comprehensively expressed in various tissues [22]. It is therefore reasonable to assume that this receptor possesses some unidentified biological functions. Indeed, GHSR1b decreases the cell surface expression of GHSR1a and acts as a repressor of the constitutive activity of GHSR1a when overexpressed in HEK-293 cells [23]. This finding indicates that GHSR1b may act as an endogenous modulator for GHSR1a constitutive activity.

Ligand binding stabilizes the active conformation of GHSR1a. The main binding pocket is deep in the cavity created by the TM domains. Both endogenous and non-endogenous ligand binding causes a conformational change in GHRS1a molecular structure characterized by a reciprocal rearrangement of the α-helices with vertical seesaw movements of TM VI and TM VII around their central proline residues. This alteration renders the intracellular ends of TM VI and TM VII to move away from the center of the receptor toward TM III, exposing the sites subsequently recognized by G-proteins and β-arrestin. The binding domain for the ghrelin is composed of six amino acids located in TM III, TM VI, and TM VII [24]. Ligand interaction with one pocket formed by polar amino acids in TM II/TM III and another formed by nonpolar amino acids in TM V/TM VI is required for binding of ghrelin with GHSR1a [18]. In contrast, the inverse agonist d-Arg^1^-d-Phe^5^-d-Trp^7,9^-Leu^11^-substance P requires a wider binding pocket, which is dispersed across the main binding crevice [19]. Studies using both peptidyl ligand GHRP-6 and nonpeptidyl ligand MK-0677 reveal Glu^124^ in the TM III domain as one of the key amino acids in the electrostatic interaction of ligand with GHSR1a [25]. Substitution of Gln for Glu^124^ in human GHSR1a eliminates its function, while mutation of Arg^283^ in TM VI disrupts its interaction with Glu^124^, and therefore abolishes both constitutive and agonist-induced signaling [26]. Disruption of the disulfide bond between Cys^116^ and Cys^198^ in the extracellular portion of GHSR1a completely abolishes the activity of all agonists [16,25]. The Glu^187^ residue in the second extracellular loop is also critical for ghrelin binding and activation of GHSR1a. Glu^187^ to Ala mutant (E187A) decreases ghrelin- and GHRP6-evoked intracellular calcium responses relative to that in the wild-type receptor [27].

Genetic analysis indicates that missense mutation of *GHSR1a* is associated with isolated GH deficiency (IGHD) and idiopathic short stature (ISS) in distinct ethnic groups such as Europeans [28], Brazilian [29] and Japanese [30]. Substitution of 611 site nucleotide from C to A, which results in protein level change in amino acid 204 from alanine to glutamate (p.A204E), has been found in patients with IGHD and ISS in France and Morocco [28]. Interestingly, presence of p.A204E appears to be accountable for the detrimental consequence in these patients and their siblings because an additional p.A204E allele correlates with a higher degree of short stature [28]. For Brazilians, Ser84Ile and Val182Ala have been identified in ISS children including a subgroup of constitutional delay of growth and puberty (CDGP) patients [29]. For Japanese, ΔGln36, Pro108Leu, Cys173Arg, and Asp246Ala mutations are identified in patients diagnosed with either IGHD or ISS [30]. Most of these mutations lead to significant reductions in cell-surface expression and constitutive activity of the GHSR1a. *In vitro* experiments using transiently transfected HEK293 cells demonstrate that the Ala204Glu mutation reduces membrane distribution, and impairs constitutive activity of GHSR1a without affecting ligand-binding activity [28]. Similar reductions in cell-surface levels and constitutive activity of GHSR1a have been observed for Ser84Ile and Val182Ala mutations [29]. Other mechanisms involve decrease in binding affinity to ghrelin and impaired agonist- and inverse agonist-stimulated receptor signaling for Pro108Leu and Asp246Ala mutations respectively [30]. All these studies indicate the clinical relevance of *GHSR1a* missense mutations with defects of growth hormone and subsequent delay of growth and puberty.

## 3. GHSR1a-Induced Intracellular Signaling and Functional Relevance

Upon binding with ghrelin, GHSR1a undergoes a profound change in the transmembrane α helices, which alters the conformation of the intracellular loops and facilitates its interaction with G-proteins. The interaction causes the exchange of GDP bound to the G protein α subunit for GTP, which activates G protein subunits and initiates various signaling responses via a series of intracellular molecules.

### 3.1. [Ca^2+^]_i_ Signaling

The well characterized signal transduction mechanism employed by the GHSR1a is the signaling pathway which leads to the hallmark increase in [Ca^2+^]*_i_*. Two mechanisms have been reported to mediate the GHSR1a-induced [Ca^2+^]*_i_* signaling: the dominant phospholipase C (PLC)/inositol (1,4,5) triphosphate (IP_3_) signaling pathway and the debated protein kinase A (PKA)/cAMP pathway. Ligand binding activates the GHSR1a, induces the dissociation of the Gα_q/11_-subunit which subsequently stimulates the production of PLC. PLC cleaves the membrane lipid phosphoinositol 4,5 diphosphate (PtdIns (4,5) P2) into IP_3_ and diacylglycerol (DAG). IP_3_ binds with IP_3_ receptor to trigger the release of calcium from stores inside the endoplasmic reticulum, which contributes to the initial rise in [Ca^2+^]*_i_*. DAG activates the protein kinase C (PKC) which inhibits potassium channels leading to membrane depolarization, subsequent opening of voltage-gated calcium channels and extracellular calcium influx [31]. In addition to this typical Gα_q/11_/PLC/IP_3_ pathway, ghrelin may also evoke the intracellular calcium signaling by an alternate pathway. In neuropeptide Y (NPY)-containing neurons, the ghrelin-induced increase in intracellular calcium concentration is dependent on calcium influx through the N-type calcium channel. These channels are activated by the cAMP-PKA signaling pathway following the coupling of the Gαs protein to the GHSR1a [32]. Consistent with these reports, studies on adenosine, once considered as a potential ligand for the GHSR1a, also suggest that GHSR1a may respond through the Gαs/cAMP/PKA signaling mechanism. Adenosine increases levels of [Ca^2+^]*_i_* independent of the concentration of IP_3_. Pretreatment with the Gαs subunit activator cholera toxin (CTX), the adenylate cyclase inhibitor MDL-12,330 A, or the PKA inhibitor H-89 blocks the effect of adenosine on GHSR1a-induced [Ca^2+^]*_i_* signaling [33]. However, other studies raise questions on the Gαs/cAMP/PKA signaling pathway employed by GHSR1a. Ghrelin alone, the endogenous ligand for GHSR1a, shows no effect on the increase in intracellular cAMP levels [30]. Conflicting results have been reported even for the original observation suggesting that ghrelin may potentiate GHRH-induced increase in cAMP. In cells co-transfected with GHSR1a and GHRH receptor, inhibition of PLC and PKC demonstrates no effect on ghrelin potentiation of the GHRH-induced cAMP increase [34,35]. The Gαs/cAMP/PKA signaling pathway employed by GHSR1a therefore remains under debate.

The most characterized physiological function employed by GHSR1a-induced [Ca^2+^]*_i_* signaling relates to the stimulation of growth hormone release. In pituitary cells, both non-endogenous and endogenous GHSR1a agonists stimulate growth hormone release in a [Ca^2+^]*_i_* dependent manner. GHSR1a-induced increase in [Ca^2+^]*_i_* may trigger the release of neurotransmitters/hormones and gene expression. In the arcuate nucleus, GHSR1a induces [Ca^2+^]*_i_* signaling in NPY neurons [32]. Instead, [Ca^2+^]*_i_* signaling employed by GHSR1a has been reported to either stimulate or attenuate insulin release in the isolated pancreatic islet cells [9,36].

### 3.2. AMP Activated Protein Kinase (AMPK) Signaling

AMPK may mediate the effect of ghrelin/GHSR1a on the regulation of energy metabolism. In the peripheral tissues, GHSR1a mediated activation of AMPK activity regulates fat distribution and metabolism in a tissue-specific manner [37]. In the rat liver, ghrelin inhibits AMPK activity to increase triglyceride content by evoking lipogenic and glucogenic related gene expression without changing the mitochondrial oxidative enzyme activities. In contrast, ghrelin reduces triglyceride content in gastrocnemius muscle by increasing mitochondrial oxidative enzyme activities through an AMPK-independent mechanism [38]. Thus, GHSR1a may induce tissue-specific changes in intracellular signaling pathways to differentially regulate mitochondrial and lipid metabolism gene expression in order to favor triglyceride deposition in liver over skeletal muscle. In the hypothalamus, ghrelin sustains NPY/AgRP neuron firing through an AMPK-dependent presynaptic mechanism [39]. This action increases food intake and thus contributes to the maintenance of neutral energy balance.

AMPK is also proposed as the critical mediator for the protective effect of ghrelin on cardiomyocytes, neurons and hepatocytes. In both the rat heart injury model induced by isoproterenol (ISO) and the tunicamycin (Tm) or dithiothreitol (DTT) evoked endoplasmic reticulum stress (ERS) models, ghrelin has been shown to protect cardiomyocytes against injury and apoptosis through a GHSR1a/CaMKK/AMPK signaling pathway [40]. In Parkinson’s disease, ghrelin enhances dopaminergic survival via AMPK mediated increase in removal of damaged mitochondria (mitophagy) which ultimately enhances mitochondrial bioenergetics [41]. While AMPK activation has been proposed to protect cells by regulating mitochondrial biogenesis and reducing reactive oxygen species production, several observations from our laboratory suggest that GHSR1a/AMPK may not be the sole pathway involved in ghrelin-induced protection of hepatocellular injury. In the mouse hepatic injury model induced by ischemia/reperfusion, ghrelin markedly attenuates up-regulation of AMPKα phosphorylation. On the other hand, ghrelin receptor gene knockout mice demonstrate a significantly higher level of hepatic AMPKα phosphorylation induced by ischemia/reperfusion injury relative to the wild-type littermates. In addition, exogenous ghrelin significantly reduces the phosphorylation of hepatic AMPKα in mice fed a high-fat diet [42].

### 3.3. PI3K/AKT Signaling

Activation of GHSR1a by ghrelin modulates insulin receptor substrate (IRS-1) associated PI3K activity and Akt phosphorylation. In hepatoma cells, ghrelin increases IRS-1 associated PI3K activity while inhibits Akt kinase activity. Alteration of PI3K/AKT signaling increases gluconeogenesis by reversing the down-regulation of insulin on phosphoenolpyruvate carboxykinase (PEPCK) mRNA expression, a rate-limiting enzyme of gluconeogenesis that catalyzes the conversion of oxaloacetate to phosphoenolpyruvate [43].

Ghrelin also stimulates the GHSR1a-dependent IRS-1 associated PI3K/Akt signaling in 3T3-L1 preadipocytes. Inhibition of PI3K activity blocks the effects of ghrelin on the proliferation and apoptosis of these cells. Furthermore, ghrelin increases both basal and insulin-stimulated glucose transport through the GHSR1a/PI3K/Akt signaling in 3T3-L1 cells. Blockade of PI3K signaling by LY294002 completely attenuates the effect of ghrelin on glucose transport [10].

As an important player in the regulation of cardiovascular functions, ghrelin has been reported to promote vascular endothelial cell proliferation, migration, survival and angiogenesis, and to inhibit cell apoptosis [13,44,45]. The underlying mechanisms may involve GHSR1a mediated activation of MAPK and PI3K/Akt signaling pathways, although a GHSR1a-independent mechanism may not be fully excluded.

### 3.4. mTOR Signaling

As an orexigenic hormone from gastric X/A like endocrine cells, ghrelin has been proposed to exercise its effects by activating the GHSR1a in the central nervous system. While multiple signaling mechanisms have been reported to be involved in the neuronal response to ghrelin, mechanistic target of rapamycin (mTOR) signaling is worth noting. Central administration of ghrelin induces a marked up-regulation of the mTOR signaling in the hypothalamic and dorsal vagal neurons, both of which are critical in the regulation of energy metabolism. In addition, central inhibition of mTOR signaling with rapamycin significantly decreases the orexigenic effect induced by ghrelin and normalizes the up-regulation of *AgRP* and *NPY* mRNA, as well as their key downstream transcription factors: cAMP response-element binding protein (CREB) and forkhead box O1 [46]. Chronic peripheral administration of ghrelin significantly increases body weight, fat mass and food efficiency in wild-type and S6K2-knockout but not in S6K1-knockout mice [47]. These observations provide the most convincing evidence that ghrelin regulates organism energy metabolism by the central nervous system involving the mTOR/S6K1 signaling pathway.

The molecular link between the GHSR1a and mTOR signaling remains to be determined. AMPK has long been considered as a negative up-stream regulator of mTOR signaling and may therefore serve as a potential molecule bridging the GHSR1a and mTOR signaling [48]. However, current observations do not fully support this notion. Both AMPK and mTOR activities are up-regulated by ghrelin in the hypothalamic neurons. These observations contradict the classical view on the negative regulation of mTOR activity by AMPK [46]. However, other studies have shown that ghrelin increases phosphorylation of hypothalamic AMPK, while it reduces phosphorylation of mTOR [49]. Given the complexity of hypothalamic networks in the maintenance of energy balance, it is not surprising that hypothalamic neurons may act differentially in response to GHSR1a activation depending on the organism energy status.

### 3.5. MAPK Signaling

In addition to the signaling pathways described above, ghrelin also regulates the proliferation and differentiation through MAP kinase (MAPK) signaling in a wide variety of cell types ranging from adrenal gland cells, myocytes, adipocytes to osteoblasts. Activation of GHSR1a stimulates the proliferation of human and rat adrenal zona glomerulosa cells through a mechanism involving tyrosine kinase-dependent MAPK p42/p44 signaling [11]. In preadipocytes, exposure to ghrelin causes a rapid activation of MAPKs, especially ERK1/2. Inhibition of MAPK signaling by PD98059, an ERK inhibitor, significantly attenuates the mitogenic and anti-apoptotic activities of ghrelin in these cells [10]. In human embryonic stem cells (hESCs), ghrelin induces cardiomyocyte differentiation from hESCs via activation of the ERK1/2 signaling pathway [50].

Multiple signaling pathways may be involved in GHSR1a associated MAPK activation. In preadipocytes, pretreatment of cells with a Gαi/o inhibitor (pertussis toxin), PKC inhibitors (staurosporine and GF109203X), or a PI3K inhibitor (wortmannin) significantly attenuates ghrelin-induced ERK1/2 phosphorylation. In hepatoma cells expressing GHSR1a, ghrelin stimulates the MAPK signaling pathway characterized by Tyr phosphorylation of insulin receptor substrate-1 (IRS-1) and binding of growth factor receptor-bound protein 2 (GRB2) to IRS-1, an upstream signaling molecule of MAPK [43].

## 4. Modulation of GHSR1a

Numerous studies suggest that both endogenous and synthetic agonists of GHSR1a could rapidly down-regulate its own receptor expression, suggesting the existence of a feedback regulation [51–53]. Injection of rat GH3 pituitary tumor cells into female Wistar-Furth rats significantly increases levels of growth hormone, which is followed by a significantly lower level of *GHSR1a* mRNA in the pituitary [52]. In *dw*/*dw* dwarf rats with growth hormone deficiency, the expression of *GHSR1a* is markedly increased in the hypothalamus, while administration of bovine growth hormone reverses this stimulation [53]. These findings suggest the presence of an intricate regulatory network governing the GHSR1a and highlight the importance of the mechanism involved in the regulation of GHSR1a in the physiological functions of ghrelin such as metabolic homeostasis, aging, immune modulation, and integration of complex physiological systems.

The regulation of GHSR1a responsiveness potentially involves molecular events governing receptor signaling, expression, desensitization, receptor interaction, and constitutive activity. These mechanisms as they pertain to ghrelin and GHSR1a are currently under active investigation.

### 4.1. Regulation of GHSR1a Expression

It is well characterized that both mRNA and protein expressions of GHSR1a are significantly down-regulated when the receptors are continuously exposed to either endogenous or synthetic agonists. These observations suggest a dynamic change in the GHSR1a expression which is subjected to the regulation of transcriptional factors and hormones.

#### 4.1.1. Regulation by Transcriptional Factors

The 5-flanking regions of the *GHSR1a* gene are structurally similar and conserved among species. It contains a TATA-less, CpG island promoter. Analysis of the 5-flanking regions in different species predicts numerous transcription factor binding sites including bHLH, AP2, and the POU-domain transcription factors Pit-1, Oct-1, and Ptx-1 which are involved in pituitary-specific expression. Amongst these transcriptional factors, Pit-1, a pituitary specific transcriptional factor, is well-characterized. A putative consensus biding site for Pit-1 has been revealed in the 5′-untranslated region of *GHSR1a* gene [54]. Promoter activity analysis also confirms Pit-1 as an important transcriptional factor in the regulation of *GHSR1a* expression. In a series of experiments in which the *GHRS1a* promoter region is inserted into a vector containing bacterial luciferase, significant expression is observed in rat pituitary cells, but not in other cell lines such as COS7 monkey kidney cells, human endometrium Skut-1B cells, mouse hypothalamic LHRH neuronal GT1–7 cells, and mouse corticotroph pituitary AtT20 cells. These results suggest a cell specific expression of *GHSR1a*. Further experiments demonstrate that Pit-1 significantly increases the luciferase activity in the monkey kidney COS7 cells, suggesting that *GHSR1a* gene expression is controlled by its binding with Pit-1 [15]. In addition, GHRH stimulates the expression of *GHSR1a* mRNA by increasing the Pit-1 level [51,55]. Studies by Soto *et al.* (1995) provide elegant evidence demonstrating an increase in transcription of Pit-1 in cultured rat pituitary cells treated with GHRH [56]. However, it is worth noting that a single dose of GHRH, which is sufficient to induce maximal release of growth hormone in porcine somatotrophs, reduces levels of *GHSR1a* mRNA by half [57]. Reasons accounting for these conflicting observations are currently unknown.

Other possible binding sites for bHLH and AP2 transcription factors have been proposed to be involved in the regulation of GHSR basal activity [54].

#### 4.1.2. Regulation by Hormones

Expression of GHSR1a is also under the control of hormones. In cultured rat pituitary cells, both β-estradiol and triiodothyronine increase levels of *GHSR1a* mRNA [15]. Further experiments using an RNA synthesis inhibitor and examination of mRNA decay rates demonstrate that the thyroid hormone up-regulates levels of GHSR1a mRNA by increasing the stability of the *GHSR1a* transcript [58]. In contrast, hydrocortisone decreases levels of *GHSR1a* mRNA by inhibiting *GHSR1a* gene promoter activity [59]. Growth hormone significantly increases levels of *GHSR1a* mRNA in the arcuate (ARC) and ventromedial nuclei (VMN), and hippocampal CA1 and CA2 neurons of GH-deficient dwarf rats. Administration of growth hormone reduces levels of *GHSR1a* to significantly below normal in ARC and VMN, suggesting a possible mechanism for feedback regulation of GHSR1a [53]. Katayama *et al.* (2000) confirms a similar increase in levels of *GHSR1a* mRNA in the pituitary gland derived from growth hormone-deficient rats [60]. Ghrelin itself also reduces the expression of *GHSR1a* in somatotrophs by 62% relative to controls [57].

### 4.2. Desensitization of GHSR1a

Desensitization is a consequence of a combination of the uncoupling of the receptor from heterotrimeric G-proteins and the internalization of cell surface receptors to intracellular compartments. Receptor desensitization provides a mechanism for protecting cells against receptor overstimulation and is commonly observed in GPCR including GHSR1a. Deficiencies in the receptor desensitization system may result in an uncontrolled or defective stimulation of target cells with consequent physiological changes. Since dissociation of the GPCR receptor from heterotrimeric G-proteins has been extensively reviewed in the literature, we focus our discussion on the internalization of GHSR1a.

The dynamics of GHSR1a internalization has been evaluated by two different assays: imaging using confocal microscopy in CHO cells stably expressing the human GHSR1a tagged at its *C* terminus with EGFP, and radioligand binding in HEK-293 cells stably expressing the human GHSR1a. Kinetic studies demonstrate that GHSR1a is internalized by endocytosis in a time-dependent manner with a peak at approximately 20 min after ligand stimulation. Internalized GHSR1a is sorted into endosomes and recycled back to the cytoplasmic membrane. The EGFP-labeled GHSR1a co-localizes with the early endosomal protein 1, an endosome marker, but not with cathepsin, a lysosomal marker. Once the ghrelin–GHSR1a complex is internalized into intracellular vesicles, GHSR1a is sorted into endosomes to be recycled back to the membrane. About 360 min after agonist removal, levels of GHSR1a on the cell surface recover almost 100%. This process is prevented by inhibitors of endosomal acidification: NH_4_Cl and concanamycin, but is not affected by inhibition of protein biosynthesis, suggesting that GHSR1a is replenished from endosomes rather than *de novo* synthesis [61]. Functional studies also support the concept of ghrelin receptor recycling. Growth hormone response to two consecutive pulses of ghrelin is significantly attenuated when pulses are separated by a short interval of 60 min, whereas growth hormone response retains its initial amplitude when the second pulse is administered after 180, 240, or 360 min [62].

GHSR1a internalization to recycling compartments depends on its *C*-terminal motifs and constitutive activity. Basal endocytosis of GHSR1a which is critical for its constitutive activity occurs without significant phosphorylation. Experiments using cultured cells over-expressing a dominant-negative β-arrestin 1 fragment (319–418) or cells derived from β-arrestin 1/2 double gene knockout mice demonstrate no alteration in basal GHSR1a endocytosis, suggesting that β-arrestin does not affect basal GHSR1a internalization. In contrast, agonist-induced internalization of GHSR1a is determined by the receptor phosphorylation and subsequent recruitment of β-arrestin proteins. Levels of GHSR1a phosphorylation are relatively low under basal conditions but significantly enhanced after ghrelin treatment. The phosphorylation site appears to be located in the *C*-terminal motif of the GHSR1a. Replacement of this domain with the GPR39 receptor *C* terminus markedly increases both basal and ghrelin-induced phosphorylation of GHSR1a relative to the wild-type receptor. β-Arrestin association desensitizes G protein-mediated signaling but also targets GHSR1a for clathrin-mediated endocytosis. This concept is supported by the observation that GHSR1a recruits the clathrin adaptor, arrestin 2, which is tagged with green fluorescent protein to allow for trafficking to endosomes after ghrelin stimulation [63].

GHSR1a internalization is also influenced by lipid and plasma membrane composition. Oligounsaturated fatty acids (OFAs) have been demonstrated to disrupt plasma membrane structure, rendering the membrane more fluid. Exposure of cells expressing GHSR1a to OFAs such as oleic and linoleic acids for a prolonged period significantly increases receptor sensitivity to ghrelin by reducing the internalization of GHSR1a [64]. On the other hand, the inhibitory effects of ghrelin pretreatment on subsequent ghrelin responsiveness are markedly blunted, suggesting that OFAs suppress desensitization of GHSR1a. All these studies indicate that the membrane composition affects GHSR1a activation and desensitization.

### 4.3. Receptor Interaction

Existence of functional GHSR1a homodimers or heterodimers has been supported by *in vitro* studies on the receptor trafficking and cellular signaling, and in one investigation by physiological experiments. Interaction between GHSR1a and other GPCRs has been extensively studied in the pituitary cells, neurons and cell lines.

Using bioluminescence resonance energy transfer methods, GHSR1a homodimers have been detected in both cytoplasm membranes and endoplasmic reticulum. Presence of sufficient GHSR1a homodimers on the cell surface may ensure the maximal responses to agonist stimulation. Instead, GHS-R1a/GHS-R1b heterodimers are concentrated within the endoplasmic reticulum. This finding suggests that GHSR1b traps GHSR1a within the endoplasmic reticulum by the process of oligomerization [65].

In the pituitary cells, numerous studies suggest that GHSR1a interacts with the GHRH receptor to enhance GHRH-induced cAMP signaling and its subsequent growth hormone release [21]. Amplification of GHRH-induced cAMP accumulation by ghrelin in the pituitary cells requires a mechanism involving GHSR1a-mediated activation of PKC [34,35]. This interaction is further validated by experiments in HEK293 cells co-transfected with GHSR1a and GHRH receptors. Ghrelin markedly increases GHRH-induced cAMP accumulation in these cells. Pretreatment with PKC inhibitor blocks the synergistic effect.

Other interactions include the adenosine receptor. Adenosine induced [Ca^2+^]*_i_* signaling is only observed in cells transfected with GHSR1a, but not in native cells. These results suggest a potential interaction between GHSR1a and the adenosine receptor. Such an interaction appears to occur at the intracellular signaling levels. Further experiments reveal that adenosine signals through adenosine receptor 2b to potentiate the GHSR1a-mediated [Ca^2+^]*_i_* signaling by a mechanism involving Gαs/cAMP/PKA mediated phosphorylation and its subsequent activation of IP_3_ receptors [33,66].

In the hypothalamic neurons, heterodimerization of GHSR1a and melanocortin-3 receptors (MC3R) causes mutual signaling interference [67]. GHSR1a significantly increases melanocortin-induced cAMP signaling, while the interaction with MC3R markedly impairs the ghrelin-induced Gαq/11 signaling and the agonist-independent basal Ca^2+^/calmodulin-induced cAMP-responsive element-binding protein signaling activity of GHSR1a. In addition, the agonist-independent basal signaling activity of GHSR1a can determine the functional signaling of the MC3R in a dimer. These findings indicate that the heterodimeric organization of two GPCRs with preferences for different G-proteins can modulate mutually and oppositely the signaling capacities of both receptors. Further investigation will reveal the importance of GPCRs dimerization in the hypothalamic control of food intake and energy homeostasis.

Formation of heterodimers of GHSR1a and the dopamine receptor has also been reported [14]. The interaction between GHSR1a and dopamine receptor subtype 1 (D1R) is supported by co-immunoprecipitation of these two receptor proteins and by functional studies. Immunoprecipitation of cell lysates from HEK293 cells expressing both GHSR1a and D1R using a GHSR1a antibody demonstrates the formation of GHSR1a/D1R heterodimers in the presence of dopamine and ghrelin. Analysis of intracellular signaling pathways reveals a switch in G-protein coupling of the GHSR1a from Gαq/11 to Gαi/s upon agonist-induced formation of GHSR1a/D1R heterodimers. When activated alone, GHSR1a predominantly couples to Gαq/11, while D1R typically signals through Gαs to activate the adenylate cyclase isozyme 2 (AC2). Upon co-activation by both ghrelin and dopamine, GHSR1a and D1R form a heterodimer that subsequently induces a conformational change in the GHSR1a. This conformational change results in coupling of GHSR1a with Gα_i_ protein, releasing βγ subunits that associate with AC2, thereby amplifying AC2 activity. Consistent with these observations, PTX treatment significantly inhibits ghrelin amplification of dopamine-induced cAMP accumulation.

Dimerization of GHSR1a and D2R has been supported by both Tr-FRET methodology and functional studies [68]. Heterodimers formed at equimolar concentrations of GHSR1a and D2R are detected by Tr-FRET assays using SNAP-GHSR1a and CLIP-tagged D2R. Interestingly, heterodimers of GHSR1a and D2R are detected not only in cultured cells but also in hypothalamic and striatal membrane preparations, suggesting the presence of endogenously formed GHSR1a/D2R heterodimers. Moreover, dopamine-induced mobilization of intracellular calcium correlates with the Tr-FRET signal produced by GHSR1a/D2R heteromers. The formation of GHSR1a/D2R dimers is relevant to the regulation of food intake and energy metabolism. Dopamine has been shown to inhibit food intake by its activation of D2R in the lateral hypothalamus. The anorexic effect of D2R activation can be blocked by either GHSR1a antagonism or gene deletion. Cabergoline, a selective D2R agonist, significantly reduces food intake relative to control animals. However, food intake in *GHSR1a* gene knockout mice is unaffected by cabergoline. Furthermore, cabergoline-induced anorexia is blocked by a highly selective ghrelin receptor antagonist (JMV2959). All these studies support the concept of physiological relevance of dimerization between GHSR1a and D2R in the hypothalamus to the energy homeostasis.

The 5-HT receptor, a centrally expressed GPCR, is also involved in satiety signaling. Heterodimers between the GHSR1a and the 5-HT2C receptor have been demonstrated. Dimerization of the GHSR1a with the unedited 5-HT2C-INI receptor, but not with the partially edited 5-HT2C-VSV isoform, significantly suppresses the agonist inducing GHSR1a mediated [Ca^2+^]*_i_* mobilization, which is completely restored after blockade of the 5-HT2C receptor [69]. While these results may suggest a potential novel mechanism for fine-tuning GHSR1a receptor-mediated activity via dimerization of the GHSR1a with other GPCRs involved in the regulation of appetite and food reward, it is worth noting that heterodimerization occurred in cultured HEK293A cells does not necessary represent the *in vivo* condition.

### 4.4. Constitutive Activity of GHSR1a

Relative to other GPCRs, GHSR1a shows an unusually high constitutive activity. Evidence is emerging that the high basal activity of GHSR1a contributes to downstream signaling and physiological processes.

The constitutive activity of GHSR1a is determined by an aromatic cluster formed by three residues (Phe VI:16, Phe VII:06, and Phe VII:09) on the inner face of the extracellular ends of GHSR1a TM VI and TM VII. It is the formation of the hydrophobic core between TM VI and TM VII that ensures proper docking of the extracellular end of TM VII into TM VI, mimicking agonist activation and stabilizing the receptor in active conformation. Specific residues in the vicinity of this cluster contribute to orchestrate microswitches critical for the activation level in absence of ligand. Amongst these surrounding amino acid residues, Trp VI:13 is crucial for the high constitutive activity of GHSR1a, because it is located in the conserved motif CWxP in the middle of TM VI and functions as a global toggle switch model allowing the inward movement of this domain [19,70,71]. Mutation of this residue (Trp276Ala) significantly impairs the ligand-independent activity. Other surrounding amino acid residues such as Val131 and Ile134 also impact the constitutive signaling of GHSR1a. Mutation of these two residues (Val131Leu and Ile134Met) dramatically increases the basal activity of GHSR1a. In addition, a mutation (Ala204Glu) in the extracellular loop II of the human GHSR1a leads to the restriction of the extracellular loop II segment and the decrease in the constitutive signaling level of GHSR1a [28,72].

Constitutive activity of GHSR1a induces both PLC/IP_3_/[Ca^2+^]*_i_* and calcium/calmodulin kinase IV (CaMK IV)/cAMP responsive element-binding protein (CREB) signaling pathways. PLC/IP_3_ signaling is the first specifically associated with the GHSR1a constitutive activity [20,73–75]. Activation of PLC and its subsequent IP_3_ production and [Ca^2+^]*_i_* mobilization are mediated via Gαq/11 protein. Constitutive activity of GHSR1a is also detected by CRE reporter gene assay and serum response element (SRE) luciferase assay [71]. CREB activation is induced through Gαs/Gαi/cAMP/PKA and Gαq/11/Ca^2+^/calmodulin-dependent kinase IV and protein kinase C (PKC) signaling pathways [76,77]. Alternatively, GHSR1a-induced SRE activity may partly be transduced by the Gα12/13–Rho pathway [71]. GHSR1a constitutive activity may be dependent on cellular context, although its underlying mechanism remains unknown. In HEK-293 cells, transfection of GHSR1a constitutively stimulates both CRE and SRE activities. However, no constitutive activity is detected in the pituitary cell line RC-4B/C40 [78].

Emerging evidences supports the physiological relevance of GHSR1a constitutive activity in growth hormone release, food intake and neural activation. The association of GHSR1a constitutive activity with PLC/IP_3_ signaling and subsequent intracellular calcium mobilization in the pituitary cells suggests a potential role in the regulation of growth hormone release. The clinical finding of missense GHSR1a mutation (Ala204Glu) in Moroccan patients supports the role of GHSR1a constitutive activity in growth hormone release. Ala204Glu-point mutation, which alters exclusively the GHSR1a constitutive activity measured by POU1F1-luciferase reporter assay, is associated with familial short stature syndrome. Lines of evidence also suggest that GHSR1a constitutive activity plays an important role in the physiological regulation of energy metabolism. Patients expressing an uncharacterized GHSR1a mutation (Phe279Leu) affecting the Phe 279 residue (Phe VI:16), a critical residue for GHSR1a constitutive activity, are characterized by increased obesity and short stature [79]. Absence of GHSR1a constitutive signaling is therefore proposed to cause a syndrome characterized not only by a short stature, but also by obesity [80]. This concept is further confirmed by the observation that intracerebroventricular injection of [d-Arg(1), d-Phe(5), d-Trp(7,9), Leu(11)]-substance P, an inverse agonist for GHSR1a, significantly decreases food intake and body weight likely by reducing gene expressions of *neuropeptide Y* (*NPY*) and *uncoupling protein 2* (*UCP2*) in the hypothalamus [81]. All these studies indicate that GHSR1a constitutive activity may provide an ultimate novel strategy for the therapy of obesity. New regulatory properties of GHSR1a constitutive activity have also been discovered in *ghrelin* or *GHSR1a* gene knockout mice. GHSR1a constitutive activity increases limbic seizures in rodents. *GHSR1a* knockout mice demonstrate a higher seizure threshold than their wild-type littermates when treated with pilocarpine. Inverse agonism and desensitization/internalization of the GHSR1a attenuate limbic seizures in rats and epileptiform activity in hippocampal slices [82]. Other studies suggest that GHSR1a activity is involved in functional impairment in learning and memory [83], hippocampal-dependent learning and habituated feeding responses [84], and arousal [85]. Further investigations are necessary to determine whether these physiological functions involve GHSR1a constitutive activity.

## 5. Summary

Studies on the GHSR1a have revealed many fundamentals on its molecular structure, intracellular signaling, constitutive activity, and interaction with other GPCRs. Many of these findings are relevant to the physiological functions of GHSR1a and of interest for translational research. Future opportunities for GHSR1a research are four-fold: (1) to define the mechanism underlying the tissue specific response in GHSR1a-mediated intracellular signaling; (2) to examine the tissue specificity of GHSR1a constitutive activity and ligand-induced response and their physiological relevance; (3) to explore the integrative function of GHSR1a in the intricate regulatory network governing energy metabolism and metabolic homeostasis; (4) to evaluate the potential applications of GHSR1a agonists, antagonists or inverse agonist in the treatment of obesity and its related metabolic diseases.
